# A method for real-time classification of insect vectors of mosaic and brown streak disease in cassava plants for future implementation within a low-cost, handheld, in-field multispectral imaging sensor

**DOI:** 10.1186/s13007-018-0350-3

**Published:** 2018-09-20

**Authors:** Joseph Fennell, Charles Veys, Jose Dingle, Joachim Nwezeobi, Sharon van Brunschot, John Colvin, Bruce Grieve

**Affiliations:** 1 School of Electrical and Electronic Engineering, University of Manchester, Oxford Road, Manchester, M13 9PL UK; 2 School of Physics and Astronomy, University of Manchester, Oxford Road, Manchester, M13 9PL UK; 3 Natural Resources Institute, University of Greenwich, Chatham Maritime, Kent, ME4 4TB UK

**Keywords:** Multispectral imaging, Real-time, Virus, Disease, Insect, Whitefly, Cassava, *Bemisia tabaci*, Begomoviruses

## Abstract

**Background:**

The paper introduces a multispectral imaging system and data-processing approach for the identification and discrimination of morphologically indistinguishable cryptic species of the destructive crop pest, the whitefly *Bemisia tabaci*. This investigation and the corresponding system design, was undertaken in two phases under controlled laboratory conditions. The first exploited a prototype benchtop variant of the proposed sensor system to analyse four cryptic species of whitefly reared under similar conditions. The second phase, of the methodology development, employed a commercial high-precision laboratory hyperspectral imager to recover reference data from five cryptic species of whitefly, immobilized through flash freezing, and taken from across four feeding environments.

**Results:**

The initial results, for the single feeding environment, showed that a correct species classification could be achieved in 85–95% of cases, utilising linear Partial Least Squares approaches. The robustness of the classification approach was then extended both in terms of the automated spatial extraction of the most pertinent insect body parts, to assist with the spectral classification model, as well as the incorporation of a non-linear Support Vector Classifier to maintain the overall classification accuracy at 88–98%, irrespective of the feeding and crop environment.

**Conclusion:**

This study demonstrates that through an integration of both the spatial data, associated with the multispectral images being used to separate different regions of the insect, and subsequent spectral analysis of those sub-regions, that *B. tabaci* viral vectors can be differentiated from other cryptic species, that appear morphologically indistinguishable to a human observer, with an accuracy of up to 98%. The implications for the engineering design for an in-field, handheld, sensor system is discussed with respect to the learning gained from this initial stage of the methodology development.

## Background

The paper reports the design of a novel instrumental technique based on active close-proximity multispectral imaging (MSI), to deliver real-time non-destructive identification of insect viral vectors. The engineering approach utilised has been designed to be translated into a rapid, portable, non-destructive and low-cost unit for use by semi-skilled field workers, notably in Sub-Saharan African countries. The focus of the investigation has been to address autonomously identifying the species and number of whitefly (*Bemisia tabaci*) insects on a leaf surface, or similar environment, in order to identify and quantify the vectors of the damaging plant viruses that cause epidemics of cassava mosaic disease (CMD) and cassava brown streak disease (CBSD) in Sub-Saharan African cassava (*Manihot esculenta*). However, the underlying technical approach may be extended by future studies to insect viral vectors of a broad range of crop, livestock and human diseases. This technology will also have applications in whitefly research generally and will, for instance, make the rearing of colonies of pure species for experimentation much less labour-intensive and expensive. As such, it is a technology that is highly appropriate for adoption in Development Assistance Committee (DAC) listed countries.

The work to-date, has been undertaken within two phases. These provide evidence as to the suitability of the technique to autonomously segment the insect vector information from within MSI datasets, with respect to their host plants. This is achieved through the modulation of narrowband semiconductor light sources, i.e. light emitting diodes (LEDs). It should be noted that throughout this paper the latter approach is referred to as multispectral, which indicates a reduced number of discrete wavebands, as opposed to hyperspectral, that commonly implies hundreds of measurements bands [1]. This is deliberate as the methodology has been developed to minimise the number of wavelengths required and, as a consequence, the ultimate system cost and complexity. Phase-1 of the study illustrated how, through coupling with a suitably modified broadband imaging detector the latter may then enable high signal-to-noise image data to be recovered such that the insect species can be classified from the picture elements (pixels). Phase-2 of the investigation then extends the concepts, by collecting a higher spatial and spectral resolution reference dataset from a broader range of insects, than in the earlier phase, and immobilising those under controlled conditions to ensure image integrity. The Phase-2 dataset includes both a wider range of vector species, as well as variations in the feeding environments of the insects. The latter is a particularly significant element of the analysis, as it points towards the utility of the tool to provide meaningful sensor data under the variations found within real-world field environments.

This research has built upon prior learning gained from insect classification using single-point vibrational spectroscopy of insect species, typically in the near to mid-infrared regions of the electromagnetic spectra. The originality of the reported work is in:

- The extension of such concepts into the visible to near-infrared bands, i.e. ~ 400–1100 nm wavelengths, *combined with*
- The utilisation of the spectral information from each pixel within a multispectral ‘datacube’ to segment the dataset into the most significant elements upon which to base the classification.

The above methodology requires the sensor system to recover the spectral information, per image pixel, at an appropriately high signal-to-noise ratio (SNR). By virtue of the availability of mass-produced, high-frame rate, sensitive silicon imaging detectors, as a consequence of the digital camera sector, such a system may now be engineered at a relatively low-cost within a handheld package.

This paper presents the laboratory evidence to support the viability of such a system design and discusses the ongoing work to realise a practical in-field engineered unit. The study to-date has been undertaken on adult whiteflies, and excludes the identification and discrimination of other life stages, i.e. crawlers, nymphs and eggs. The extension to include the latter is theoretically achievable and the approach is particularly aligned to delivering that due to its ability to extract the insect tissue elements from within the background host leaf structure.

### Importance of *B. tabaci* to cassava and global development

Cassava is a uniquely important food-security crop for one-third of the world’s low-income, food deficit countries and is the world’s third largest source of calories in the human diet [2]. After 15 years of global increases, however, cassava production is predicted to fall due to droughts and diseases. CMD and CBSVD in particular, destroy an estimated 35 million tonnes of African cassava annually [2]. *B. tabaci*, and the greater than 200 plant-viruses it transmits, have an undeniable impact on the world’s food security, directly affecting a broad range of staple crops such as cassava, common bean and sweet potato. In Africa, over 200 million people obtain close to 50% of their daily food intake from cassava [2].

### Disease management programmes for *B. tabaci*

Once the viral vectors are identified within a region management programs may be implemented to control the spread of begomoviruses. For example, in the 1990s a CMD pandemic caused widespread famine in East Africa. This was driven by unusually high *B. tabaci* populations generated, in part, by a virus-vector-host plant interaction that increased *B. tabaci* fecundity on CMD infected cassava, boosting vector numbers and driving disease spread [3]. The disease was contained through developing and distributing CMV-resistant cassava varieties to farmers and the variety TMS 30572 (Migyera) was adopted rapidly and more than 11,338 ha were being grown by 1996 [4]. However, in more recent years resistant strains of the virus have resulted in a continued spread of CMD and CBSD across Africa [5] requiring further containment and eradication programs to be instigated.

### Motivation behind sensor development for in-field viral vector identification

*Bemisia tabaci* is not a single species [6], but consists of a group of more than 34 closely-related cryptic species. At present, these can only be identified using molecular markers. Although Africa is the evolutionary origin of *B. tabaci*, its diversity there remains poorly studied and so the number of species will continue to increase as more are identified. Several African *B. tabaci* species feed on cassava and the rapid spread of CMD and CBSD has been associated with high whitefly populations of the species now called, SSA1 and SSA2. As well as *B. tabaci*, other whitefly species such as *Bemisia afer* also colonise cassava [7] and due to their similar size, they can be confused easily with *B. tabaci* [8]. Current survey and epidemiological research in sub-Saharan Africa focussed on *B. tabaci* borne viruses relies on destructive sampling of the subjects followed by a multi-step molecular sequencing technique, of partial mtCO1 sequences, to identify the whitefly species present. The costs and time taken to undertake the necessary steps for this diagnostic method are often prohibitive, when thousands of individual whitefly need to be typed accurately. The complexity of the analysis also necessitates that it is undertaken within centralised laboratory facilities using comparatively expensive instrumentation, with the corresponding delays in analytical lead times and the requirements for servicing and support infrastructure.

As a consequence, with existing technology, it is not possible to do detailed landscape ecological research in cassava-based agro-ecosystems, because of the magnitude of the species identification problem. The delivery of the real-time, portable and non-destructive sensor system, as introduced within this paper, would enable the spread of insect viral vectors to be mapped in a reliable and timely manner. This may be achieved by exploiting the sensor data alongside the accurate positional and time data as derived from a linked smartphone handset and then using the latter to relay just the relevant extracted information to a central data-base, so minimising bandwidth usage. Furthermore, the approach proposed may be undertaken rapidly by semi-skilled field operatives, as it requires no pre-treatment of the leaf or insect samples. Such a tool offers the potential for virologists and plant breeders to deliver a paradigm shift in the eradication of CMD and CBSD, through targeted control measures and introduction of new varieties, with the consequent impact on cassava production in Africa and broader implications for deploying variants of the technology-platform for viral disease control elsewhere.

### Prior research for rapid assessment of viral vectors

Previous research has been undertaken to identify the symptoms of CMD and CBSD, on cassava leaves, through visual imaging algorithms that are compatible with the modest processing power within mobile phone handsets [9, 10]. More recently it has been reported that similar approaches have been adapted to count the numbers of *B. tabaci* on the underside of cassava leaves (Mwebaze, E., Makerere University, Kampala, Uganda; unpublished) but, due to the morphological similarity of the species, such techniques have not so far been capable of identifying the separate *B. tabaci* species from their visual images. An additional problem in getting accurate population counts of African cassava *B. tabaci* nymphs arises in the field, because they are pale and silvery in colour and so do not stand out against the green leaf background.

Autonomous morphological classification techniques have been applied to invertebrates from colour images, e.g. [11]. However, the variation in orientation, illumination and viewing angle from field-captured images of insects, as opposed to the larger vertebrates, combined with the lack of significant morphological differences between *B. tabaci* species, prevents their usage within this duty. Instead greater success has been achieved through the application of near to mid-infrared linear models (1.4–2.1 µm wavelength). These have previously been developed for autonomous species identification of the age and/or species in entomology, such as: mosquitos [12, 13], flies [14], beetles [15], termites [16], wasp pupae [17], psocids [18], worms [19] and ants [20]. These models have been developed using single-point conventional laboratory spectrometers, which provide a composite measure of the infrared-active organic functional groups within the beam of the spectrometer, e.g. the C–H, N–H, O–H, C–C or S–H atomic bonds within the molecular groups on the surface of the pests [12]. However, such instrumentation requires comparatively exotic semiconductor materials, tending to be based upon indium gallium arsenide (InGaAs) detectors, as proprietary, low-cost, silicon detectors are limited to a wavelength upper limit of 1125 nm, due to the bandgap of silicon. This compares to a similar wavelength limit for InGaAs of 3540 nm.

### Rationale for undertaking the research

As minor pigment variations between the whitefly species have been observed under laboratory conditions the hypothesis was that detection of species differences could be automated using a miniaturised MSI systems, combined with morphological and spectral processing of the data. The resulting system could then be exploited in the field to identify and count autonomously, in real-time and non-destructively, known viral-vectors from their non-virus-carrying neighbours.

An existing prototype MSI instrument, which was developed primarily for the commercial arable agricultural industry was adapted for the whitefly duty. This system utilises active, close proximity, MSI based on proprietary components from the consumer electronics industry. This is based upon narrowband LEDs which are then integrated with modified colour silicon-CMOS imaging detectors, such that their spectral range is extended from the visible (Vis) region (400–700 nm wavelengths) out into the near infrared (NIR), up to the bandgap of silicon (wavelengths up to 1125 nm). Such an approach offers the potential to deliver cellular/wireless network-connected handheld sensors for in-field use at US$10–100’s as opposed to the non-portable laboratory near to mid-infrared equipment used in the previous entomological species identification work which cost in excess of US$30,000 (2016) [10–18].

The ability to use whole image rather than gross single-point spectral measurements also opens up the possibility of greater discrimination accuracy, without the need for skilled preparation of the insect samples. The latter is by virtue of being able to use combined image and spectral feature extraction to automatically extract the insect features from the background, correct for insect orientation and then use parts of the structure of the insect, as opposed to the whole body, to provide specific species identifier markers.

This technology has to-date been developed with industrial partners, for duties ranging from protein assessment, weed control and early detection of fungal diseases [19]. Through a ratio analysis of images taken under far-red and infrared illumination [20] non-vegetative material, within the images, may be simply extracted from the data allowing more detailed spectral analysis to be undertaken on the remainder.

This paper is differentiated from the prior-art as it exploits both the spatial and spectral data from actively illuminated viral vectors to generate significantly greater differentiation of the insects, and their eggs, from the background leaf structure. It further enhances the in-field capabilities from such a unit by generating spectrally-enhanced images, using linear multivariate analysis techniques, which have been demonstrated to be capable of distinguishing species of whitefly, and potentially other insects, that are known viral vectors (carriers) of CMD and CBSD, versus their benign counterparts. This is based on low-cost, miniaturised and low-power commercial electronic components operating across the Vis to NIR wavelengths of light.

## Methods

### Phase-1 trial

The first phase of study utilised a prototype 63 waveband, 380–940 nm wavelength, prototype bench-top MSI unit so as to image the relatively small whitefly features (c.1–2 mm in length) versus the larger supporting leaf structure [21]. This system also enabled the much smaller nymphs (c.500 µm) and eggs (c.100 µm) to be imaged. The latter are extremely challenging to identify by eye within the fibrous texture of cassava leaves even with the aid of a lab microscope.

The LEDs within the unit were conventional sources providing non-coherent light across wavelength range of 365–940 nm at 5–10 nm Full Width Half Maximum (FWHM) resolution, in a virtually continuous coverage of the spectra with the exception of the 530–585 nm bands. The latter was due to the lack of availability of suitable LEDs at the time. Subsequently this range, as well as that from 940 to 1050 nm, has been included within the prototype unit, but such systems post-date the trials reported within this paper. LED control and image capture was achieved through a proprietary single board computer (Raspberry PI Model 2, Raspberry PI Foundation, UK), interfaced to a bespoke driver card. The approach provided a cost-effective and flexible platform, for initial proof-of-concept studies, prior to developing a more optimised portable dedicated unit for rapid in-field measurements. The spectral output and illumination power of each of the LEDs was characterised using a laboratory spectrometer (Ocean Optic USB4000, Ocean Optics Inc., USA) and the intensity balanced, across the wavelength range, using Pulse Width Modulation of the drive signals. A barium sulphate reference tile was used to calibrate the spectral intensity of the light output from each of the LEDs.

This Phase-1 study comprised ten whole leaf samples of cassava and eggplant (*Solanum melongena*), with 30–70 adult whitefly on each leaf (both male and female). These had been removed from healthy plants that had previously been exposed to pure colonies of four cryptic species of African *B. tabaci*, reference: SSA1-SG1, SSA2, SSA3 and MED Uganda ASL, see Table 1, the first three being CMD insect vectors and the last is not. After removal the leaves were chilled, so as to partially immobilise the insects. This was necessary for these preliminary studies as the prototype MSI instrument was not optimised for rapid image capture and so required between 2 and 5 min, per leaf, to accumulate the necessary spectral images and then store them to memory.

**Table 1:** Details of laboratory colonies of cryptic *B. tabaci* species

*Bemisia tabaci* cryptic species designation	Year; location of collection	GenBank accession number
*B. tabaci* Sub-Saharan Africa 1 subgroup 1 (SSA1 SG1)	2015; Port Harcourt, Nigeria	MG565970
*B. tabaci* Sub-Saharan Africa 2 (SSA2)	2013; Kiboga, Uganda	MG565971
*B. tabaci* Sub-Saharan Africa 3 (SSA3)	2015; Nkalagu, Nigeria	MG565972
*B. tabaci* Mediterranean Uganda ASL (MED ASL)	2013; Gayaza, Central Uganda	MG565977
*B. tabaci* Mediterranean Q1 (MED Q1)	2013; Malaga, Spain	MG565975
*B. tabaci* Sub-Saharan Africa 1 subgroup 3 (SSA1 SG3)	2013; Tanzania	MG565976
*B. tabaci* Asia II 1 (Asia II 1)	2013; Lodhran, Pakistan	MG565973
*B. tabaci* New World 2 (NW2)	2013, Brazil	MG565974

The table include the suspect viral vectors species designation, its sample date and location and the specific accession number in the GenBank

The raw multispectral image data were processed using the Optimised Soil Adjusted Vegetative Index approach [20] to mask-out the background supporting leaf structure. This was selected as the inclusion of a scaling parameter (r = 0.16) in the denominator reduces the influence of incident light reflected from non-plant surfaces onto the leaf surface. The MSI data was then further processed to exclude the smaller items (speckles) which were predominantly a consequence of specular reflectance errors. The resulting multispectral data were then autoscaled (mean centred and each variable scaled to unit standard deviation) and processed using the supervised multivariate classification technique, Partial Least Squares Discriminative Analysis (PLS-DA), by using 20 randomly selected samples of each species of whitefly as the training set. This, and the pre-conditioning of the data, was undertaken using custom code written within the MatLab environment (MatLab R2015a, Mathworks, USA).

### Phase-2 trial: *B. tabaci* species and experimental host plants

Prior to the second phase of trials in May 2017, laboratory colonies of five different cryptic species of *B. tabaci*, see Table 1, were reared and maintained separately on a range of experimental plant hosts. These included: (1) cassava (*Manihot esculenta* cv. MCol22; *Euphorbiaceae*), (2) eggplant (*Solanum melongena* cv. Black Beauty; *Solanaceae*), (3) kale (*Brassica oleracea* cv Dwarf Green Curled; *Brassicaceae*), and (4) sweet pepper (*Capsicum annuum* cv. California Wonder; *Solanaceae*). The ability of each cryptic species to reproduce and develop on each host differed (due to known variation in host-plant range). Due to their inability to survive, colonies could not be established for every *B. tabaci*/experimental host-plant combination. Due to fluctuations in colony size, 1 population of the *African cassava mosaic virus* (ACMV) vector (SSA1 SG3) and 2 non-vectors of ACMV (MED Q1 and Asia II 1) were present in large enough numbers (> 100 insects) to establish a reliable training and testing set. In total, 7 combinations of insect and diet were present in large enough numbers for use in the classification process. The species purity of colonies was confirmed by standard molecular methods [22]. In brief, the 3′ partial mitochondrial cytochrome oxidase 1 gene of ≥ 3 individuals of each cryptic species of *B. tabaci* was sequenced (haplotype Gene Bank accessions are provided in Table 1, column GB). The experimental *B. tabaci* species were reared on eggplant for several generations prior to the experiments, to ensure they were ACMV-free (eggplant is not an ACMV host). Their virus-free condition was confirmed by their inability to infect healthy cassava with ACMV. The *B. tabaci* colonies were reared under standard conditions of 28 ± 2 °C, 60% humidity and a 14:10 h light:dark cycle, respectively.

### Phase-2 trial: sample preparation

Due to the partial immobilisation of the whitefly on cooling during Phase-1, combined with the higher heat levels from the halogen source and the longer scan times of the Headwall instrument, an alternate technique was applied during Phase-2. Cohorts of adult whiteflies (mixed gender, different ages) were collected from each colony using custom-made aspirators which transfer whiteflies directly into separate 1.5 mL Safe-Lock micro-centrifuge tubes (Eppendorf, Hamburg, Germany). Tubes were immediately flash-frozen in liquid nitrogen and maintained under liquid nitrogen until use. Three alternative methods for killing whiteflies for analysis were trialled, including conventional slow-freezing to − 20 °C, exposure to tri-chloromethane vapour and submersion in 90% ethanol. These methods, however, damaged the specimens significantly (unpublished data).

Microscopy viewing arenas consisted of standard plastic petri dishes (60 mm internal diameter) with custom-made black rigid plastic stages inserted. Microcentrifuge tubes were removed singly from liquid nitrogen and frozen whitefly tipped onto the stage. Whiteflies settled with a mostly uniform positioning (dorsal surface facing upwards), but with differing side-to-side orientations.

### Phase-2 trial: measurement instrumentation and protocol

**Fig. 1:**
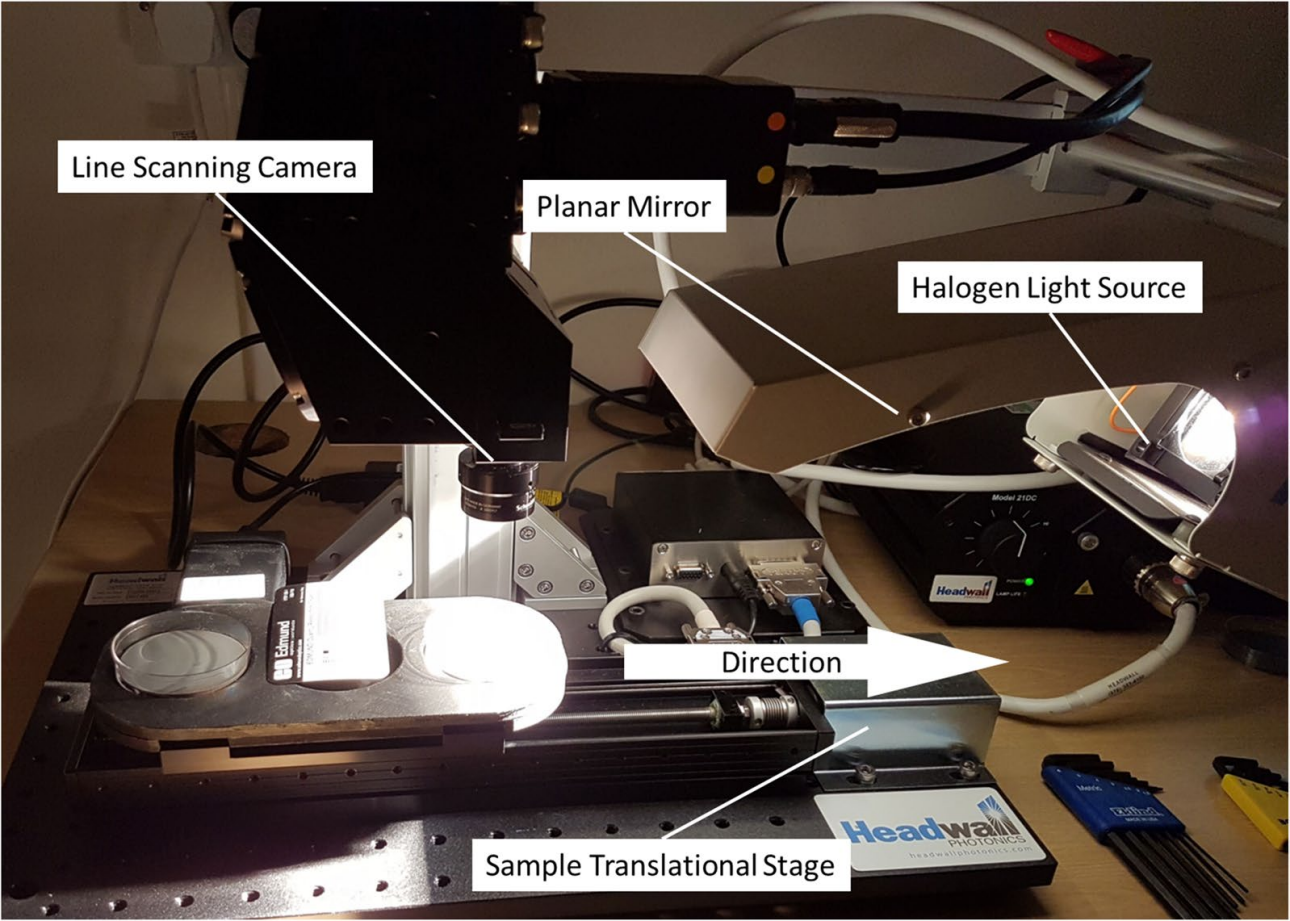
Phase-2 trial, instrumental arrangement. The figure shows the line-scanning MSI imaging unit left of centre of the image with the halogen source and mirror assembly to the right. The custom petri dish holder is below the MSI detector and includes one petri dish, incorporating the grey card background, next to the calibration card for the instrument

A commercial line-scanning MSI imaging unit was used (1003B-10143, Hyperspec^®^ VNIR A-Series, Headwall Photonics Inc., Massachusetts, USA), capable of operating across a wavelength range of 380–1000 nm with a spectral resolutions of 0.74 nm. This was fitted with a 23 mm focal length lens (1004A-21445 VNIR lens, Headwall Photonics Inc., Massachusetts, USA). The instrument uses a halogen source for illumination, which is reflected via a concave mirror to reduce the lux intensity variation across the sample. This was operated at ~ 60% of power with the distances from: lens-to-sample = 70 mm, mirror-to-sample = 150 mm and mirror-to-centreline-of-lens = 80 mm. With respect to Fig. 1, this provided a compromise between SNR achievable from the instrument whilst minimising the potential for heat damage of the whitefly.

The whitefly samples were located in a custom laser cut mount, which accepted up to three petri dishes along the direction of travel of the line-scanner’s linear motorized stage. Into the base of each dish was inserted an optically neutral ‘grey-card’ disc, again laser cut, upon which multiple immobilised whitefly were located. The exact number being dependent upon the survival rate of the various species when reared on the differing host plants, between 5 and up to 50. The resulting arrangement provided a field of view of the width of the petri dish with a spatial resolution of 60 microns. The speed of the linear stage being then adjusted to generate square aspect ratio pixels. The system was calibrated for variations in the spatial light intensity, using a white barium sulphate optical reference tile.

### Phase-2 trial: spatial image handling and segmentation method

All analyses in Phase-2 were written using the Python-3 programming language, utilising the ‘SciKit-Learn’ [23] toolbox (www.scikit-learn.org) with ‘NumPy’ [24] (www.numpy.org) to handle large array operations. The imaging system outputs flat binary files with associated header files. In addition to this, colour (RGB) preview images were saved during the experimental image capture phase. Large areas of the master MSI frames did not contain any insects, and so to reduce processing times a rapid cropping stage was used to extract the relevant portions of the data from the raw MSI files, which were in excess of 16 GBytes in size. These cut-out portions of the MSI data typically contained between 1 and 20 individual whiteflies. In total 566 cut-outs were used for training and testing. These were then saved in the same format as the master images and named with the whitefly category encoded in the filename.

**Fig. 2:**
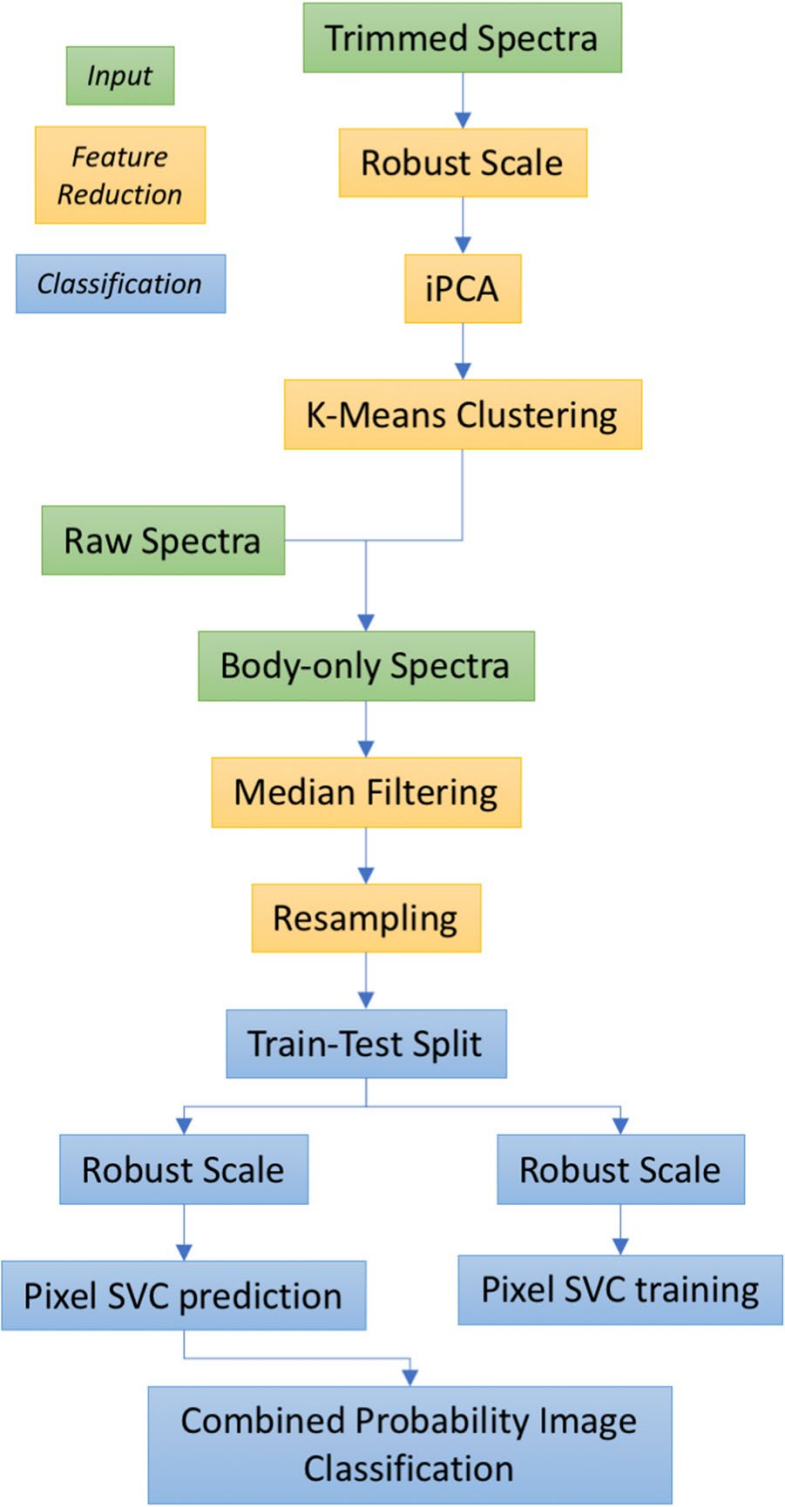
Flowchart of Phase-2 processing. The flowchart depicts the steps undertaken to process the MSI data and classify the viral vectors. The green boxes represent the input data streams: trimmed spectra—low noise spectral data, i.e. 900–1030 nm wavelengths in this case; raw spectra—data as taken from the instruments; body-only spectra—as extracted from the spatial data. The yellow and blue boxes depict the feature reduction and classification stage respectively. Abbreviations used: incremental Principle Component Analysis (iPCA), Support Vector Clustering (SVC)

With reference to the flowchart of Fig. 2, to separate different body components, across all species the spectra were clustered using unsupervised classification. Manual inspection of the images showed increased image noise below 420 nm and between 900 nm and 1030 nm. The dataset was trimmed to exclude bands in these regions as the Principal Component Analysis (PCA) method is sensitive to Gaussian noise, which reduces the robustness of the resulting classification. The robust scaling algorithm (sklearn.preprocessing.RobustScaler), from the ‘SciKit-Learn’ package, was used to mean-centre each raw pixel spectrum. In order to process the large dataset (800,000 observations of 700 spectral bands) Incremental Principal Component Analysis (iPCA—sklearn.decomposition.IncrementalPCA) was performed on the dataset. Each pixel was treated as a separate observation, i.e. spatial information not incorporated into the model.

The spectra were clustered into broad classes, using the K-Means clustering algorithm (sklearn.cluster.KMeans, ‘SciKit-Learn’) on the decomposed dataset. We hypothesised that each pixel in the cleaned dataset would be a member of either ‘wing’, ‘body’ or ‘background’ and so set the number of clusters a priori to three. All other parameters in the clustering function were held at the default values. Images were checked manually to identify the anatomical clustering. These steps can be considered a semi-supervised segmentation tool, preventing the need for manual annotation of the images to identify the body region.

As the objective of this study was to classify insect species, the body pixels identified in the previous step were used for training a support vector classifier (SVC). This uses the support vector machine algorithm implemented as a classifier (as opposed to a regressor in Support Vector Regression).

## Results

### Phase-1 trial

This initial study indicated that an 85 to 95% correct classification for whitefly species may be achieved for the four species studied, with respect to the others, using just 11 of the available 63 wavebands. The chosen wavelengths being selected based upon their relative contribution to the model (loadings) so as to achieve the highest classification accuracy for the data-set without including additional noise from non-contributing wavebands. The Phase-1 trial was limited to adult whitefly all of a similar age and cultivated under controlled laboratory conditions with similar diets and environments. However, it provided adequate evidence as to the effectiveness of using active MSI for extracting *B. tabaci*, and other leaf borne pests, from the host plant images to lead into the Phase-2 investigation. Furthermore it demonstrated that through spectral analysis of only the pixels corresponding to the pests the technique provided adequate information within the image date across the 380–980 nm wavelength range to classify *B. tabaci*, and so verified the viability of using proprietary, low-cost, silicon sources and detectors for this type of duty.

The initial trails also showed that the optical arrangement within the test instrumentation resulted in reduced image fidelity, and potential errors in species classification, associated with specular reflectance and inhomogeneous illumination of the leaf samples. The latter aspects of the system design have now been addressed, through exploiting a variant of system design for plant leaf analysis. However, pending the realisation of the revised active MSI system, the Phase-2 trials were undertaken with a high spectral resolution commercial reference hyperspectral imaging unit.

### Phase-2 trial: image segmentation and semi-automated pixel labelling

**Fig. 3:**
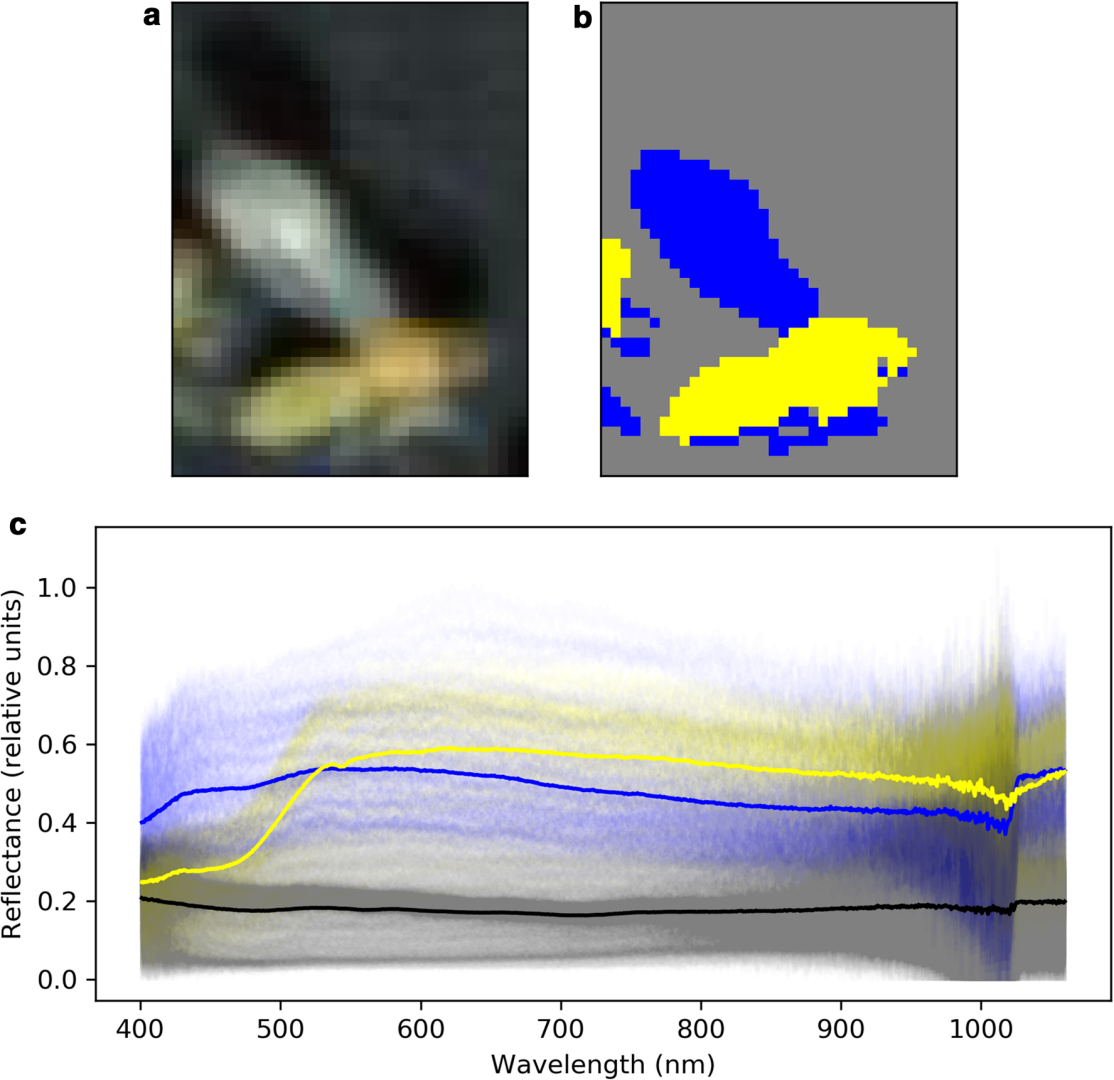
Example body region segmentation. **a** RGB image, **b** K-means classified image, **c** spectra for each pixel. In (**b**, **c**), yellow corresponds with insect body, blue with wing and grey with background. In the spectra plot, the solid lines show the class mean

The first 3 principle components explained 99.0% of the variance in the data (see Fig. 1) and so only the first 3 principle components were used for K-means clustering. Classes were colour-coded and manually inspected in a random subset of the data. A typical example is presented in Fig. 3b where the three classes can be seen to broadly corresponded to insect body, insect wing and background. From this, a class which described the insect body pixels was selected and the array describing the centroid of each of the 3 classes was stored to remove the need for manual identification of each class every time the code was run. As such, this allowed rapid identification of body pixels in our dataset, preventing the need for manual labelling of body regions in every image.

### Phase-2 trial: multivariate classification of spectral data

**Fig. 4:**
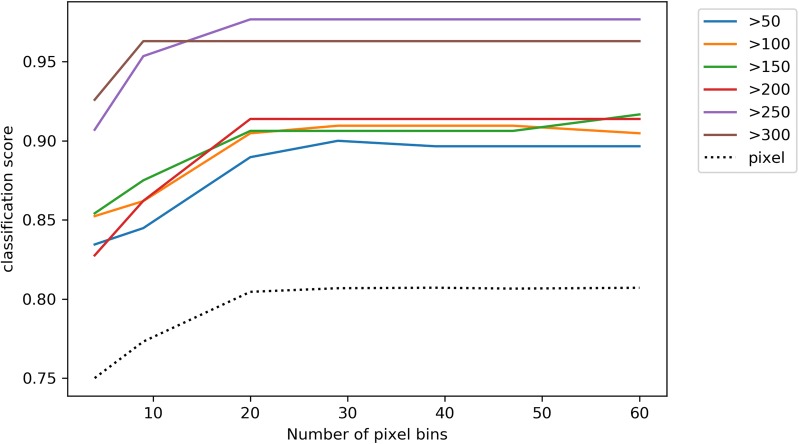
Accuracy of classifier for different degrees of spectral smoothing. The classification score is the proportion of images correctly classified, except for the line labelled ‘pixel’, which represents the proportion of pixels correctly classified. For each degree of smoothing, the minimum number of pixels per image for inclusion in the scoring is shown by different coloured lines

Only the insect body pixels, segmented as described above, were included in the next stage of analysis as these body regions where expected to be important in spectrally differentiating the species types. Each pixel spectrum was median filtered (scipy.signal.medfilt, ‘SciKit-Learn’) and resampled to between 4 and 60 equal wavebands to both reduce the dimensionality of the dataset and better represent the sensor information likely to be acquired from future, bespoke instrumentation. The data were then labelled as either ‘SSA1’ or ‘Other’. Two-class support vector classifiers (SVCs—sklearn.svm.SVC, ‘SciKit-Learn’) were then fit to ~ 50% of the data and scored against the remainder. Data were split such that subset images were not split across training and test sets. For testing, all body-pixels were classified by the trained SVC classifier, giving a pixel classification accuracy (Fig. 4). The median probability of class membership was used to determine the final classification for any given image, i.e. for a given image, if *Median* (*P*(1)) > 0.5 then Class = 1.

With reference to Fig. 4, pixel classification accuracy increased with the number of features between 4 and 20 features, however above this number there was no improvement in classification with increased numbers of features. For image classification, images with 50–200 pixels classified with similar accuracy, i.e. 89–91% correctly classified for a 20 feature classifier. However, increasing the minimum threshold to 250 pixels/image improved the classification accuracy to 98% with the same 20 feature classifier. Overall, the optimal classifier had 20 wavebands and required a minimum of 250 pixels to classify at an accuracy of 98%.

**Fig. 5:**
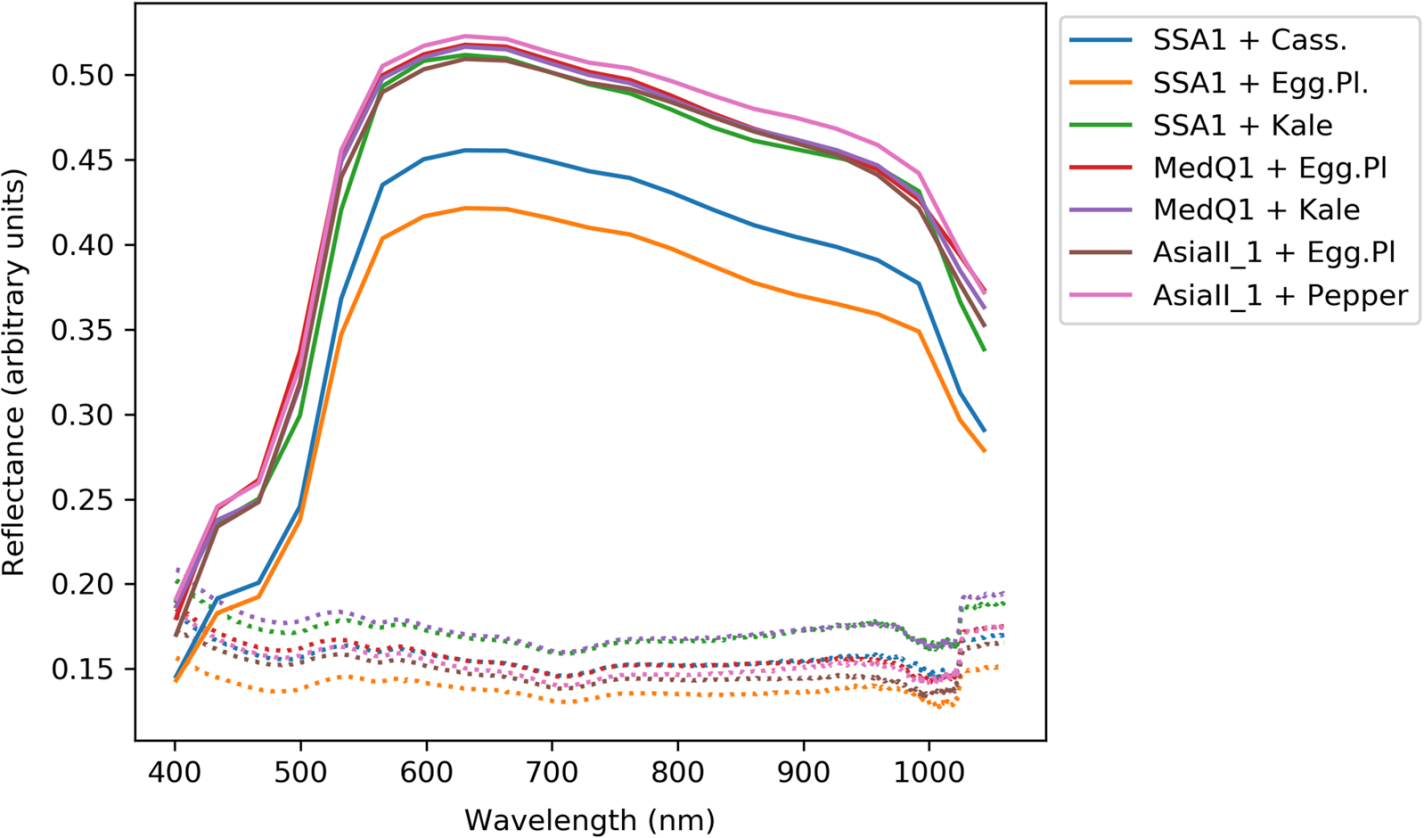
Mean spectra for different image segments for insect-by-diet combinations. Solid lines = body region, broken lines = background

Background pixel reflectance was relatively uniform across all diet-by-species classes indicating that there was no variation in illumination intensity between different image captures. With reference to Fig. 5, the biggest differences in spectral reflectance were between SSA1 SG3, as reared on cassava and eggplant, and the other classes. *B. tabaci* Asia II 1and MED Q1 had similar reflectance spectra, independent of diet. SSA1 reared on Kale appeared similar to Asia II 1 and MED Q1.

## Discussion

### Body reflectance spectra for *B. tabaci* identification

For the first time, this initial study demonstrates that through an integration of both the spatial data, associated with the multispectral images being used to separate different regions of the insect, and subsequent spectral analysis of those sub-regions, a key viral vector (SSA1 SG3) can be differentiated from other cryptic species, that appear indistinguishable to a human observer, with an accuracy of up to 98%. Findings were consistent between the two phases which showed that populations of SSA1 (subgroups 1 and 3) could be separated from populations of MED (ASL and Q1) using different instrumentation and classification methods. Furthermore, in the case of insects reared on eggplant, SSA1 SG3 had different body reflectance spectra to Asia II 1 and MED Q1 when reared on the same diet, as illustrated in Fig. 5. This strongly suggests species-specific body pigmentation occurs independently of plant-host diet.

Encouraging as the above evidence is, the study was based on a limited range of *B. tabaci* cryptic species, feeding and environmental conditions. Although the classifier performed well overall using insects reared on different diets, body pigmentation could be influenced by the host plant and this may affect classification accuracy. The large spectral differences between SSA1 SG3 fed on the three different diets, i.e. see Fig. 5, suggest that this does indeed influence body colouration and so further work is needed to understand the effect this may have on a classification system. However this aspect may be readily accommodated within a future study through a more extensive training and validation set of sampled insects, which fully reflect the breadth of host plants where the SSA1 SG3 species may be commonly found.

### Justification for an imaging system

An important feature of this work is the use of a multispectral imaging sensor rather than taking a single point spectrum. Both the first and second phase of the reported work illustrate the importance of the spatial element of the multispectral dataset, both for extracting the insect elements of the data from the host leaves (Phase-1) and then to recover the degree of spectrally relevant pixels only from the image-stack, in this case from the non-wing region of the whiteflies (Phase-2), in order to attain the necessary high degree of species classification, using extremely weak and convoluted molecular harmonic vibrations found in the Vis to NIR region of the spectrum. Single point spectral analysis in these bands would result in ‘noise’ being added to the spectral data from non-relevant areas of the sample, e.g. plant material, dust, spoil debris, insect wings or legs, in the case of a defocused reflectance-spectra measurement point, or inadequate SNR to gain the classification within appropriate time, for a focused spectral measurement point covering just a small circular area of the relevant insect body part(s). Hyperspectral imaging approaches have been used to classify closely-related species [25, 26] and different morphs of the same insect species [26], however our approach builds on these through the use of a morphological segmentation pre-processing step. This reduced the noise from non-classifiable body regions, such as the wings, and allowed robust identification of the viral vector SSA1 SG3. Using an imaging sensor also allows population estimation on individual plants through counting individuals [7, 8]. This could be important both for farmers in monitoring infestation levels and calculating insecticide dose, as well as for the breeding, epidemiology and virology research community, in seeking to understand host diversity for different whitefly species as well as to map and control the spread of the vectors across geographic regions.

### Implications for sensor system design

The instruments used within this study are not currently field-compatible, in terms of speed, functionality or cost and so we propose the development of new instrumentation based on the findings presented in this paper. In Phase-2, simulating different bandwidth sensitivity showed that a minimum of 20 wavebands, each with a bandwidth of approx. 35 nm, covering the 400–1100 nm region would be needed for optimum classification accuracy using this technique. Phase-2 findings also suggest a spatial resolution allowing at least 250 pixels per insect would be needed in order to classify at 98% accuracy. As such, future instrumentation should be designed with appropriate spatial and spectral resolution.

This study demonstrates that cryptic species identification is possible using an imaging system based on low-cost and readily available commercial silicon sources and imaging arrays, combined with relatively modest processing power. In addition to a low cost sensor, there is a requirement to implement the homogenous and diffuse (shadow free) optical illumination platform, such as reported elsewhere [27], in order to utilise the more subtle species classifier models of the Phase-2 study within the actively illuminated imaging sensor system architecture of the Phase-1 investigation. Furthermore, to enhance the usability of the system compensation is required for the orientation and distance of the focal plane of the sample with respect to the imaging array lens, across the multiple wavelengths. Similarly, the reliance on chilling of the insects to reduce their mobility and potential for reorientation during multispectral imaging has been reduced within the design for the next generation of the ‘Bravo’ unit. This exploits a dedicated FPGA, based on the Altera Cyclone V family of processors, and custom electronics for high-speed data processing and interfacing to a large-format (16 mm square) full-frame image capture sensors (Sony Pregius IMX250). Additionally, significantly higher intensity and homogeneous lighting is achieved using a combination of an optical integrating hemisphere with appropriately located LEDs driven by high power current sources, run at very short duty-cycle to prevent overheating of the sources. The resulting system reduces the capture time for a typical 32 waveband multi-spectral dataset to less than a second, whilst achieving a resolution of 2048 by 2048 pixels for each wavelength image.

## Wider context and further work

A methodology such as that presented in this paper, when translated into handheld sensor system, has the potential to offer much-needed monitoring of food security threats in Sub-Saharan Africa, especially when integrated with other meta-data streams. The projected low-cost and portable nature of a handheld unit based on readily-available components would enable such a system to be viably deployed to a large number of areas for real-time mapping of potential insect disease vectors.

As well as offering in-field diagnostics for farm management, the geographic spread and prevalence of known disease-carrier species would be dynamically mapped on a seasonal basis. Through combination with data from an on-board GPS module and direct or proxy, e.g. via Bluetooth to a cellular phone, wireless connectivity to the external world such networked multispectral sensors may act as direct or pseudo, via back-to-base downloads, real-time feeds to a Geographical Information System (GIS), for disease forecasting and mapping.

Such GIS mapping would be reliant on the access to both the above data as well as additional meta-data on crop, soil, environment and weather conditions, many of which would be derived from remotely sensed aerially or satellite derived information. The latter opens up the potential for the future handheld MSI systems to act as ground-truthing data in support of the remotely sensed disease, and other biotic and abiotic, crop stress models. In the case of CMD, CBSD and sooty moulds, such systems would appear to be viable to support direct autonomous detection of the early symptoms of the diseases directly upon cassava leaves [28], from Sentinel 2 and other publicly available satellite data sources.

## Abbreviations

ACMV: *African cassava mosaic virus*
CBSD: cassava brown streak disease
CMD: cassava mosaic disease
DAC: Development Assistance Committee
FAOSTAT: Food and Agriculture Organization of the United Nations Statistics
FWHM: full width half maximum
GB: GenBank
GIS: Geographical Information System
iPCA: Incremental Principal Component Analysis
InGaAs: indium gallium arsenide
LEDs: light emitting diodes
MSI: multispectral imaging
NIR: near infrared
PCA: Principal Component Analysis
PLS-DA: Partial Least Squares Discriminative Analysis
RGB: red green blue
SNR: signal-to-noise ratio
SVC: support vector classifiers
Vis: visible
